# Cellular Prion Protein as a Receptor of Toxic Amyloid-β42 Oligomers Is Important for Alzheimer’s Disease

**DOI:** 10.3389/fncel.2019.00339

**Published:** 2019-07-30

**Authors:** Yuan Zhang, Yanfang Zhao, Lei Zhang, Wanpeng Yu, Yu Wang, Wenguang Chang

**Affiliations:** ^1^Institute for Translational Medicine, Qingdao University, Qingdao, China; ^2^School for Life Science, Institute of Biomedical Research, Shandong University of Technology, Zibo, China

**Keywords:** cellular prion protein, amyloid-β, oligomers, receptor, Alzheimer’s disease

## Abstract

The pathological features of Alzheimer’s disease (AD) include senile plaques induced by amyloid-β (Aβ) protein deposits, neurofibrillary tangles formed by aggregates of hyperphosphorylated tau proteins and neuronal cell loss in specific position within the brain. Recent observations have suggested the possibility of an association between AD and cellular prion protein (PrP^*C*^) levels. PrP^*C*^ is a high affinity receptor for oligomeric Aβ and is important for Aβ-induced neurotoxicity and thus plays a critical role in AD pathogenesis. The determination of the relationship between PrP^*C*^ and AD and the characterization of PrP^*C*^ binding to Aβ will facilitate the development of novel therapies for AD.

## Background

Alzheimer’s disease (AD) is a progressive neurodegenerative disorder representing the most common cause of dementia in the elderly. The hallmarks of AD include senile plaques induced by amyloid-β (Aβ) protein deposits, neurofibrillary tangles formed by aggregates of hyperphosphorylated tau proteins and neuronal cell loss in specific position within the brain (Di Lazzaro, 2018). Soluble Aβ and prefibrillar oligomers have been recognized as early and key factors in AD-related synaptic dysfunction. It had initially been suspected that Aβ plaques are directly toxic to neurons, however, studies have revealed that the level of Aβ plaque deposits is not closely associated with the severity of AD (Selkoe, 2011). However, recent studies have shown a strong correlation between the oligomeric forms of Aβ and neurotoxicity and the severity of cognitive impairment in AD (Benilova et al., 2012; Kayed and Lasagna-Reeves, 2013).

The physiological prion protein (PrP^C^) is an evolutionarily highly conserved protein that is present in all investigated mammals. PrP^C^ is attached to the outer surface of the cell membrane by a glycosylphosphatidylinositol (GPI)-anchor. PrP^C^ is a glycoprotein that expressed in the brain and was first reported to be associated with prion diseases (Singh and Udgaonkar, 2015). Recent observations have indicated the possibility of a connection between prion diseases and AD (Onodera, 2017). PrP^C^ mediates at a certain extent of the toxic effects of Aβ oligomers and thus plays an important role in AD pathogenesis (Salazar and Strittmatter, 2017; Brody and Strittmatter, 2018; Purro et al., 2018). PrP^C^ is a high affinity receptor for oligomeric Aβ, and the expression of PrP^C^ is important for Aβ-induced neurotoxicity, as demonstrated by the loss of long-term potentiation (LTP) and memory impairment in AD mouse models (Gimbel et al., 2010; Kostylev et al., 2015). PrP^C^ deficiency confers resistance to the synaptic toxicity of oligomeric Aβ in mice and *in vitro* in hippocampal slice cultures (Barry et al., 2011). These findings support the hypothesis that the interaction between PrP^C^ and Aβ is necessary for neurotoxicity and neuronal cell loss in AD.

## Changes in PrP^C^ Involved in AD Pathology

### Characteristics of Distinct PrP^C^ Isoforms in AD Brains

**FIGURE 1:**
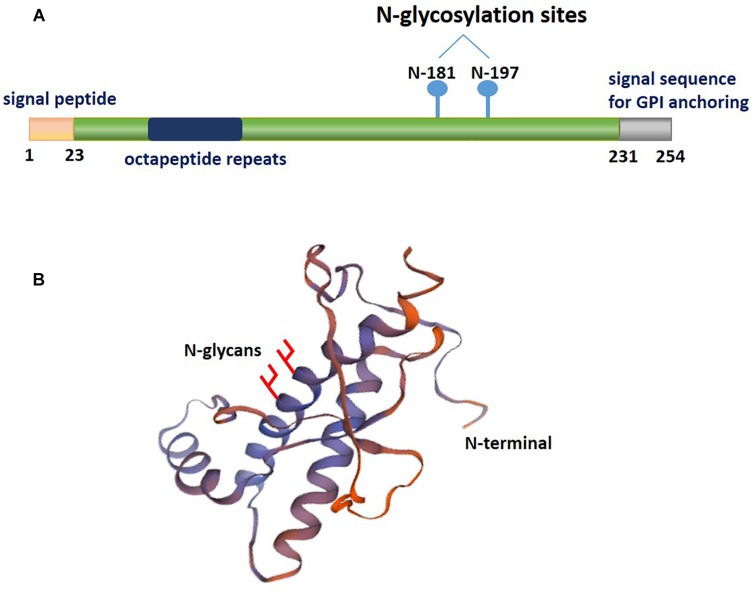
A schematic illustrating the posttranslational modifications of PrP^C^. The relative size and localization of N-glycans in a schematic representation of the structural domains of PrP^C^. **(A)** The structure of PrP^C^ can be divided into two distinct domains: a disordered N-terminal domain and an α-helical C-terminal domain. The N-terminal domain include a positively charged region at the N-terminus that is important for the endocytosis of PrP^C^, octapeptide repeats that allow PrP^C^ to bind ions, and a hydrophobic tract. The C-terminal domain consists of three α-helices and two short β-strands. This domain is also the site of posttranslational modifications in PrP^C^; up to two N-glycans are added to the α-helical domain, and a GPI anchor at the C-terminus attaches PrP^C^ to the outer surface of the plasma membrane. **(B)** The three-dimensional structure of residues 90-31 of recombinant human PrP^C^ (PDB #2lsb.1.A), as determined by NMR spectroscopy.

PrP^C^ is an N-glycosylated GPI-anchored protein usually present in lipid rafts, which is variably glycosylated at two highly conserved sites, namely, the asparagine resides at positions 181 and 197 of the human PrP^C^ (Figure 1). Distinct phenotypes and binding domains are characterized by specific mechanisms during AD progression. N-glycan attachment to these sites in PrP^C^ results in diglycosylated, monoglycosylated and unglycosylated isoforms (Wiseman et al., 2015). The ratios of diglycosylated, monoglycosylated and unglycosylated PrP^C^ have been shown to be variable in AD brains.

Velayos et al. (2009) found that the unglycosylated isoform predominates in AD patients, unlike in controls, indicating a shift in the profile of PrP^C^ glycosylation in AD pathological progression. Saijo et al. (2011) also observed significantly higher levels of unglycosylated PrP^C^ in the temporal cortex of amnestic mild cognitive impairment (aMCI) patients, but not of mild AD (mAD) patients, compared to that of NCI and AD patients. The higher levels of the unglycosylated isoform compared to the other isoforms in AD patients supports the hypothesis that the presence of certain glycosylation forms has a critical role in AD.

Additionally, Zafar et al. (2017) demonstrated a significant 1.2-fold decrease in the levels of diglycosylated PrP^C^ isoforms in rapidly progressive AD (rpAD) patients compared to controls but significantly increased total PrP^C^ levels in slow progressive AD (spAD) and rpAD patients. This study indicates that posttranslationally modified PrP^C^ isoforms are changed in the different pathological processes of AD, revealing that the different phenotypes of PrP^C^ may be risk factors for the slow or rapid progression of AD pathology.

Extensive structural analysis has shown that the unglycosylated fragment of residues 90–231 PrP^C^ lacks the flexible N-terminal part of the protein (Wuthrich and Riek, 2001). Studies have indicated the energetic stabilization of the structural region of PrP^C^ in residues 127–227 due to the consequence of the presence of an N-glycan at Asn^197^. In contrast, an N-glycan at Asn^181^, which is located in a stable secondary structure, does not influence PrP^C^ conformation but may play a functional role (Zuegg and Gready, 2000; Ermonval et al., 2003). Thus, this evidence indicates that distinct PrP^C^ isoforms are involved in the association of altered PrP^C^ interacting proteins with AD pathology. The glycosylation pattern of PrP^C^, which may become a potential diagnostic biomarker for pathology, is related to the severity of AD.

### Altered Levels of PrP^C^ Involved in AD Pathology

PrP^C^ play an important role in the pathogenesis of AD. Some pathological evidence indicates that PrP^C^ deposits often accompany Aβ plaques in AD (Schwarze-Eicker et al., 2005; Takahashi et al., 2011). The importance of the association between PrP^C^ and Aβ is greatly strengthened when it was demonstrated that PrP^C^ was the receptor for the high affinity to Aβ42 oligomers on cells (Purro et al., 2018).

Reported data suggest a regulatory influence of PrP^C^ expression in the pathological process of AD. The altered expression of PrP^C^ in aging and the development of AD are associated with disease progression, and it has been observed that PrP^C^ is decreased in the hippocampus and temporal cortex in aging and sporadic AD but not in familial AD, suggesting that PrP^C^ expression reduced reflects a main mechanism of disease and is not merely a minor consequence of other AD-associated changes (Whitehouse et al., 2010). In a study by Velayos et al. (2009), there was a tendency for a lower expression of PrP^C^ in AD patients than in healthy patients, which indicated that existing PrP^C^ expression may play a protective role in AD.

In addition, other studies have focused on PrP^C^ expression level alterations in advanced stages of AD, mainly stage Braak III to VI, most likely due to neuronal loss. Vergara et al. (2015) demonstrated that, in AD patients with Braak stages I–VI, PrP^C^ protein expression in the brain increases in the early stages of AD and peaks at approximately stage III. Thereafter, PrP^C^ expression decreases until the manifestation of clinical symptoms in both cases (Vergara et al., 2015).

However, there are some conflicting results regarding the elevation of membrane-binding PrP^C^ levels in brain tissue of AD patients compared with that of patients with mild cognitive impairment (MCI) or no cognitive impairment (NCI) (Larson et al., 2012; Beland et al., 2014). There have also been some studies showing that there is no significant diversity in the expression level of PrP^C^ between AD patients and healthy people (Saijo et al., 2011; Dohler et al., 2014; Abu Rumeileh et al., 2017). The discrepancy may due to the lack of specificity of the assay for the prion protein. Diglycosylated fragments overlap with mono- and nonglycosylated forms of full-length PrP^C^, which may potentially affect the quantification of PrP^C^ levels and explain the discrepancy. As Saijo et al. (2011) further demonstrated, there were no differences in total PrP^C^ levels, however, the glycosylated forms were observed to be significantly changed in AD.

These studies reinforce the hypothesis that changes in PrP^C^ levels are critical for AD pathological development. Clarifying the possible relationship between cognitive decline, PrP^C^ expression and differentially glycosylated PrP^C^ is extremely important for the identification of AD.

## PrP^C^ Mediates Neurotoxicity by Aβ Oligomers

### Regions of PrP^C^ Involved in Binding to Aβ

The crucial role of PrP^C^ in neurodegeneration, especially in AD, is complex. The distinct functions of the various domains of PrP^C^ have been associated with determining the early trigger of AD pathophysiology. The amino-terminal octapeptide repeat domain of PrP^C^ (resides 60–95) participates in extracellular copper ion binding (Viles et al., 1999; Jackson et al., 2001). The unstructured central domain of PrP^C^ (residues 95–134) contains a charge cluster (residues 95–110) and a segment with hydrophobic character (residues 112–134), which has been implicated in playing a critical role in neurodegenerative activity (Baumann et al., 2007; Li et al., 2007; Lauren et al., 2009).

Several independent studies have shown that the interaction of Aβ oligomers with the N-terminal residues of the PrP^C^ protein region appears critical for neuronal toxicity (Lauren et al., 2009). The PrP^C^-Aβ42 interaction provides important mechanistic insights into the pathophysiology of AD-related neurodegeneration. Dohler et al. showed that PrP^C^-Aβ binding always occurs in AD brains and is never detected in nondemented controls and that the binding of Aβ aggregates to PrP^C^ is restricted to the N-terminus of PrP^C^ (Dohler et al., 2014). Antibodies targeting PrP^C^ N-terminal residues can prevent synaptic plasticity deficits induced by Aβ oligomers (Didonna et al., 2015). For instance, Lauren et al. (2009) found that the deletion of residues 32–121 of PrP^C^ abrogates binding to Aβ42 oligomers, indicating that the globular domain alone cannot mediate the role of binding to Aβ. The hydrophobic region of PrP^C^ (residues 105–125) is not an essential determinant for binding activity due to this region binds Aβ42 oligomers in a way that is indistinguishable from its binding to full-length PrP^C^ and further the Δ32–106 variant was similar to the Δ32–121 variant, having no Aβ42-oligomer affinity (Lauren et al., 2009). Pretreatment with an antibody against residues 93–109 of PrP^C^, 6D11, can prevent neuronal cell death by oligomeric Aβ42 and rescues cognitive deficits in APP/PS1 transgenic mice, while another antibody against residues 144–152 of PrP^C^, 6H4, fails to block oligomeric Aβ-induced neuronal toxicity (Chung et al., 2010; Kudo et al., 2012). Recently, another antibody against resides 23–111 of PrP^C^ was reported to rescue synapses and cognitive deficits in APP/PS1 mice (Cox et al., 2019).

The addition of a synthetic peptide of PrP^C^ residues 98–107 can reduce the neurotoxicity of Aβ oligomers in primary hippocampal cells, whereas the addition of a peptide of PrP^C^ residues 213–230 has no effect on Aβ-induced neurotoxicity (Kudo et al., 2012). Blocking residues 95–105 of PrP^C^, but not its C-terminal residues, can effectively prevent the inhibition of LTP (Barry et al., 2011; Larson et al., 2012). In addition, Chen et al. indicated that both N-terminal residues 23–27 region and the 92–110 region, which are critically important for PrP^C^ interactions with Aβ42 oligomers because the deletion of either of these regions results in a major loss of binding, are highly flexible and natively unstructured (Chen et al., 2010). Zou et al. demonstrated three Aβ42-specific PrP^C^ peptide regions, including N-terminal residues 47–59, 53–65, and 87–99, as well as three Aβ42-nonspecific peptides, including residues 25–37, 37–49, and 99–111 from the N-terminal domain of PrP^C^. These Aβ42-specific binding sites are localized in the octapeptide repeat region of the unstructured N-terminal domain. The regions have important implications regarding the pathophysiological consequences of Aβ-PrP^C^ interactions (Zou et al., 2011).

**FIGURE 2:**
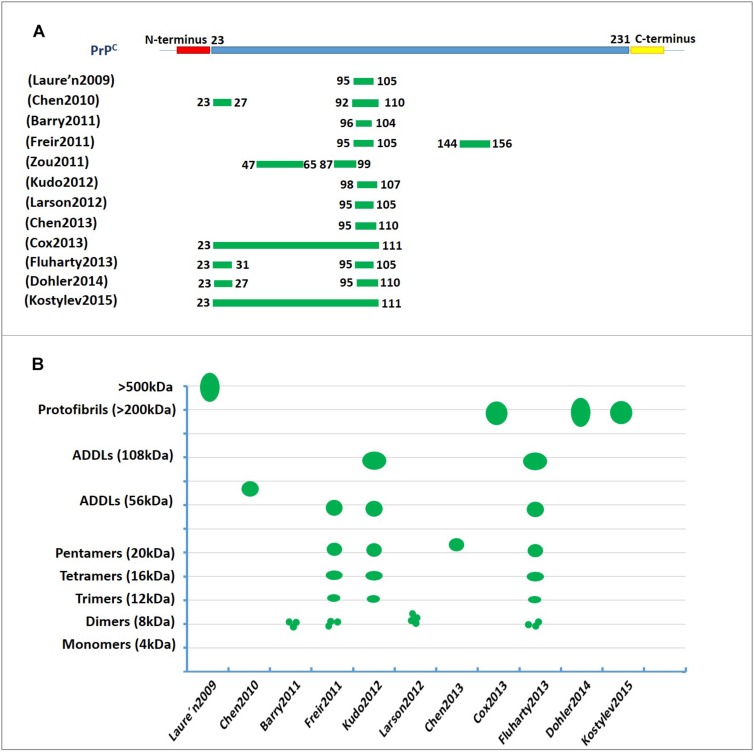
The characterization of PrP^C^ binding to Aβ42. **(A)** The regions involved in PrP^C^ binding to amyloid-β (Aβ). The green lines represent the amino acid sequence of PrP^C^ involved in binding to Aβ. The residue 95–110 region of PrP^C^ contains the critical amino acid sequence for binding oligomeric Aβ. **(B)** A diagram depicting the binding of amyloid-β (Aβ) in various aggregation states to PrP^C^. The green represents the molecular weights of Aβ assemblies bound to PrP^C^ published in previous studies. The Y-axis represents the molecular weight of the various Aβ42 oligomers. ADDL, Aβ-derived diffusible ligand.

Collectively, these studies strongly suggest that N-terminal residues 23–27 and the 95–110 region of PrP^C^ contain the critical amino acid binding sequence for oligomer Aβ-induced synaptic impairment and neuronal cell death (Figure 2A and Table 1). These soluble recombinant PrP proteins and their fragments are strong inhibitors of the cytotoxic and synaptotoxic effects of Aβ42. The identification of specific huPrP regions that are crucial for the interaction with Aβ may also contribute to the development of therapeutic strategies for targeting this interaction.

**TABLE 1:** The characterization of PrP^C^ binding to Aβ42.

**Studies**	**The positions of PrP**^C^ **binding to Aβ**	**The forms and sources of Aβ42**		**Detection methods**
Lauren et al., 2009	PrP^C^ 95–105	Oligomers (∼500 kDa)	Synthesized	SEC
Chen et al., 2010	PrP^C^ 23–27, PrP^C^ 92–110	Oligomers (>75 kDs)	Synthesized	AFM
Barry et al., 2011	PrP^C^ 96–104	Dimers (8 kDs)	Brain-derived	WB
Freir et al., 2011	PrP^C^ 95–105, helix-1 (PrP^C^ 144–156)	ADDLs (8∼56 kDs)	Synthesized/brain-derived	SEC
Zou et al., 2011	PrP^C^ 47–59, PrP^C^ 53–65, PrP^C^ 87–99	Oligomers	Brain-derived	SEC
Kudo et al., 2012	PrP^C^ 98–107	ADDLs (trimer∼24 mer, 108 kDa)	Synthesized	AFM/WB
Larson et al., 2012	PrP^C^ 95–105	Dimers (8∼9 kDa)	Brain-derived	SEC
Chen et al., 2010	PrP^C^ 95–110	Oligomers (>25 kDs)	Synthesized	TEM/ WB
Cox et al., 2019	PrP^C^ 23–111	Globulomer AβO (∼200 kDa)	Brain-derived	SEC
Fluharty et al., 2013	PrP^C^ 23–31, PrP^C^ 95–105	ADDLs (Dimers 8–108 kDs)	Synthesized	SEC
Dohler et al., 2014	PrP^C^ 23–27, PrP^C^ 95–110	Dimers to pentamers (8∼21 kDa)	Synthesized	SEC
		150∼300 kDa	Brain-derived	
Kostylev et al., 2015	PrP^C^ 23–111	Globulomer AβO (∼200 kDa)	Brain-derived	SEC

**Summary of publications identified the regions of PrP^*C*^ binding to Aβ42, and the sources and aggregation states of Aβ42 used. AFM, Atomic Force Microscope; SEC, Size exclusion chromatography; TEM, Transmission Electron Microscopy; WB, Western Blot; ADDLs, Aβ-derived diffusible ligand.**

### PrP^C^-Aβ- Binding Assemblies

For decades, research on the molecular mechanism of AD has focused on the compositions of the plaques that are one of the characteristics of the disease (Gouras et al., 2010, 2015). In recent years, it has been found that low molecular weight Aβ aggregates form as Aβ oligomers, and highly structured protofibrils have now emerged as the key neurotoxins in AD (Sakono and Zako, 2010; Brouillette, 2014).

Membrane-binding PrP^C^, as a receptor for Aβ oligomers, has been demonstrated to be involved in regulating LTP in the hippocampus, which is induced by oligomeric Aβ42. Recently, it was found that PrP^C^ can interact not only with the oligomeric form of Aβ, but also with other forms. For example, synthetic Aβ oligomers, Aβ-derived diffusible ligands (ADDLs) and soluble extracts from AD brains have been reported to interact with PrP^C^, yet all of these forms of Aβ are unsuccessful at abolishing LTP in PrP-null mice (Freir et al., 2011). Zou et al. (2011) demonstrated that recombinant human PrP (huPrP) also exhibits high affinity and specificity for Aβ42 oligomers from brain extracts of AD patients, and recombinant huPrP may represent an intrinsic molecular spectrum of PrP^C^ *in vivo*. The inhibition of LTP by human brain extracts containing dimeric amyloid-β is prevented by an antibody fragment (Fab) directed to PrP^C^ *in vivo* in rats (Barry et al., 2011). Further findings indicate that fragments of the PrP^C^ protein can prevent deficits in synaptic plasticity and neuronal death induced by toxic dimers and trimers of Aβ oligomers species (Scott-McKean et al., 2016). The above studies have revealed that the interaction of certain amyloid species with PrP^C^ leads to neuronal degeneration, and these Aβ species are oligomeric forms with low molecular weights.

Recently, studies have indicated that neurotoxins comprise high molecular weight Aβ assemblies, referred to as ADDLs, which are found to impair synaptic plasticity and memory dysfunction in AD. PrP^C^ has also been shown to bind to ADDLs, which are tightly related with cognitive impairment in multiple mouse models of Alzheimer’s disease (Fluharty et al., 2013; Kostylev et al., 2015). Freir et al. demonstrated that PrP^C^ is a major component for the inhibition of LTP by ADDLs from AD brains, which is consistent with research showing that oligomeric Aβ assemblies bind with strong specificity to PrP^C^ to trigger the disruption of synaptic plasticity *in vitro* and *in vivo* (Freir et al., 2011; Kostylev et al., 2015).

However, a separate study indicated that PrP^C^ shows strong binding to high molecular mass assemblies of Aβ (158–300 kDa) derived from the brains of Alzheimer’s disease patients, but not to small synthetic oligomeric Aβ42 (Dohler et al., 2014). It is possible Aβ protofibrils or high molecular mass assemblies correlate better than either globular oligomers or amyloid fibrils with PrP^C^ binding. High molecular mass assemblies are not simply chains of globular oligomers, but instead contain a defined triple helical nanotube structure that may relate to their specific PrP^C^-dependent toxicity (Nicoll et al., 2013; Purro et al., 2018). It is possible that the connections of Aβ42 and PrP^C^ are not exclusive, and other sterically similar β-sheet rich assemblies might interact with and signal through PrP^C^ (Resenberger et al., 2011).

Thus, PrP^C^ appears to be important to mediate the plasticity impairments induced by certain Aβ species or conformations that must be clarified in the future. Figure 2B and Table 1 show the molecular weights and aggregation states of Aβ42 binding to PrP^C^ currently. Dimers, ∼108 kDa oligomers and ADDLs display a much higher ability to interact with PrP^C^.

### Characterization of Soluble and Insoluble Aβ in AD

The major components of Aβ aggregates that form in AD brain are neuritic plaques, diffuse amyloid, and vascular amyloid. A variety of other assemblies including Aβ protofibrils and soluble oligomers of various sizes have also been identified (Caughey and Lansbury, 2003; Haass and Selkoe, 2007). Evidence suggests that Aβ oligomers are soluble and may spread throughout the brain, yet amyloid fibrils are larger and insoluble and may assemble into amyloid plaques (Ferreira et al., 2015). However, the present findings strongly support that soluble Aβ oligomers are more detrimental to synaptic plasticity.

Aβ oligomers are categorized by molecular weight as low-molecular-weight (LMW) oligomers ranging from dimers-tetramers and high-molecular-weight (HMW) oligomers ranging from ∼50 to 150 kDa (Figueiredo et al., 2013). A recent study indicated that LMW and HMW oligomers have differential binding affinities for neurons and neurotoxicity. LMW oligomers acutely impair synaptic plasticity, whereas HMW oligomers induce neuronal oxidative stress via activation of NMDA receptors (Wisniewski et al., 2011).

However, other studies have shown that both soluble and insoluble fractions of brain homogenates bind to PrP^C^ in transgenic mouse models of AD (Zou et al., 2011; Larson et al., 2012). Dohler et al. (2014) demonstrated that optimal binding to PrP^C^ occurs in the insoluble fraction of Aβ. Their data show that Aβ is present as insoluble oligomers in all tested high molecular weight fractions. Another study using AD and healthy brains also showed the preferential binding of high molecular weight Aβ42 assemblies to PrP^C^, which occurs mainly in the insoluble fraction of Aβ, in AD (Zou et al., 2011).

In fact, the composition, concentration and purity of Aβ samples from AD brains are always different. For synthetic Aβ42, small oligomeric species show prominent binding to PrP^C^, whereas in AD brains larger protein assemblies containing Aβ42 bind efficiently to PrP^C^ (Dohler et al., 2014). Furthermore, the natural separation of different Aβ aggregates present in AD brain samples is complex because preparation methods can disrupt and alter the conformation of Aβ assemblies (Stine et al., 2011). Synthetic Aβ is generally soluble, while the Aβ extracted from AD brains may contain both soluble and insoluble fractions (Table 1). Some soluble oligomers may bind to other macromolecules or to cell membranes and can therefore become insoluble (Dohler et al., 2014).

In addition, Wildburger et al. (2017) recently found 26 unique proteoforms in soluble and more insoluble Aβ aggregates of AD brain samples, including 73% N-terminal truncations and 30% C-terminal truncations of the total Aβ proteoforms. The Aβ proteoforms segregated between the soluble and more insoluble aggregates, with N-terminal truncations predominating in the insoluble material and C-terminal truncations segregating into the soluble aggregates. This result suggests that the Aβ aggregates in AD are heterogeneous and offers much new evidence for investigation into the pathological mechanisms of AD.

## Molecular Consequences of the PrP^C^/AβO Interaction in AD

The molecular and cellular consequences of the PrP^C^-Aβ oligomer interaction are dependent on raft-based complexes. The integrity of cholesterol-rich lipid rafts is critical for the interaction between Aβ42 with PrP^C^. It has been demonstrated that the PrP^C^-mediated toxicity of Aβ oligomers and the activation of downstream pathways require lipid rafts (Rushworth et al., 2013). GPI-anchored PrP^C^ is localized to the cholesterol-rich lipid raft microdomains of the plasma membrane (Taylor and Hooper, 2006). Cholesterol depletion disrupts these rafts with PrP^C^ being redistributed into nonraft regions of the membrane (Taylor et al., 2005). A study revealed that the disruption of the rafts causes a significant reduction in Aβ oligomer binding to cells and prevents the activation of Fyn kinase (Rushworth et al., 2013).

A growing body of evidence suggests that PrP^C^ mediates downstream intracellular processes through many different receptors, including the metabotropic glutamate receptors mGluR1 and mGluR5, the α7 nicotinic acetylcholine receptor, the kainite receptor GluR6/7, and AMPA receptor subunits GluA1 and GluA2 (Zhao et al., 2010; Carulla et al., 2011; Jeong and Park, 2015; Haas and Strittmatter, 2016). These studies indicate that PrP^C^ functions as an extracellular scaffolding protein that is able to organize multiprotein complexes that mediate intracellular signal transduction at the cell surface.

A separate study implicated group I mGluR signaling in the regulation of Aβ42 toxicity in neurons and in AD mouse models, and Aβ42 oligomers interact with PrP^C^ to increase metabotropic glutamate receptor 5 (mGluR5)-dependent long-term depression (LTD) and LTP (Hu et al., 2014). Another study showed that the role of the Aβ/PrP^C^ complex in AD pathophysiology; the complex has been demonstrated to induce intracellular Fyn activation, leading to the further phosphorylation of the NR2B subunit of the N-methyl-D-aspartate (NMDA) receptor and to the destabilization of dendritic spines (Um et al., 2012; Brody and Strittmatter, 2018). In addition, Pyk2 has been reported to be a downstream effector of PrP^C^-Aβ signaling. This study revealed Pyk2 as a direct tyrosine kinase of tau that is active downstream of Fyn (Li and Gotz, 2018).

Recently, the Aβ-PrP^C^ complex was reported to be internalized into endosomes via low-density lipoprotein 1 (LRP1) in AD. Rushworth et al. (2013) revealed that PrP^C^-mediated binding and toxicity and the subsequent PrP^C^-mediated internalization of Aβ oligomers are dependent upon LRP1. Research has indicated that LRP1 functions as a transmembrane coreceptor that is involved in the PrP^C^-mediated binding of Aβ oligomers (Rushworth et al., 2013).

The laminin receptor (LRP/LR) has been demonstrated to play a significant role in the interaction between Aβ and PrP^C^, given that the laminin receptor binds and internalizes PrP^C^. The blockade of LRP/LR ameliorates the detrimental effect of PrP^C^ overexpression on cell viability upon exposure to exogenous Aβ (Pinnock et al., 2016). The binding of Aβ to PrP^C^ leads to the induction of apoptosis by interacting with LRP/LR, which, as a transmembrane receptor, may influence the activity of the JNK signaling pathway. However, the proapoptotic signals pathways may not be directly transduced by PrP^C^, as PrP^C^ is not a transmembrane protein and therefore must be transduced through other receptors binding to PrP^C^, such as LRP/LR (Da Costa Dias et al., 2014).

**FIGURE 3:**
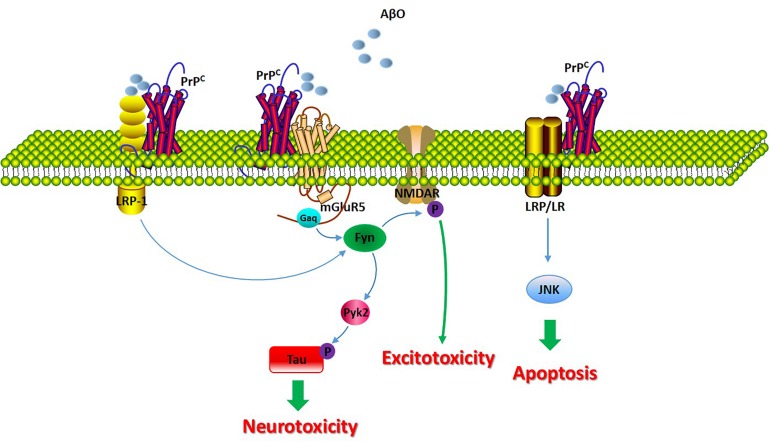
The molecular consequences of the PrP^C^/Aβ interaction in AD. The signaling pathway involved in the PrP^C^/Aβ interaction in AD. The PrP^C^/Aβ complex can interact with mGluR5, LRP1, and LRP/LR. The PrP^C^/Aβ complex induces Fyn activation, leading to the further phosphorylation of the NR2B subunit of the NMDA receptor and to the destabilization of dendritic spines. Additionally, Fyn activation of Pyk2 leads to the further phosphorylation of tau. LRP1 functions as a transmembrane coreceptor that is involved in PrP^C^/AβO-mediated Fyn activation. LRP/LR is a transmembrane receptor involved in the apoptotic signaling pathway through interactions with PrP^C^-Aβ.

Thus, further investigations have been made to expound these complex and their downstream pathways to prevent neurotoxic consequences (Figure 3).

## Conclusion

Currently most researches show that PrP^C^ may play an important role in the pathogenesis of AD. There is a connection between AD and PrP^C^ levels. PrP^C^, as a high affinity receptor for oligomeric Aβ and ADDLs (8∼108 kDa), is essential for Aβ-induced synaptic toxicity. N-terminal residues 23–27 and the 95–110 region of PrP^C^ contain the critical amino acid sequence for oligomer Aβ-induced synaptic impairment and neuronal cell death.

However, there is some controversy about the neurotoxicity of PrP^C^. Some literature reports that PrP^C^ deletion does not inhibit the toxic effects of Aβ oligomers. For example, Balducci et al. (2010) demonstrated that Aβ oligomers induced *in vivo* memory impairment and bound PrP^C^ with high affinity but found that PrP^C^ is not responsible for the recognition impairment in AD induced by Aβ. However, others have reported that PrP^C^ is a key receptor mediating toxic effects, but these assumption have not been confirmed (Larson et al., 2012). Perhaps PrP^C^ is not the unique receptor that mediates synaptic damage induced by Aβ oligomers. PrP^C^ has been involved in neurotoxic signaling with high-affinity binding to Aβ oligomers, suggesting that the interaction of PrP^C^ with Aβ is part of a common molecular pathway. The most recent investigations indicated the existence of a strong interaction between Aβ oligomers and PrP^C^ and suggest that this interaction may impact synaptic plasticity functions. Further investigations will be necessary to clarify the involvement of PrP^C^ in the neuropathology of AD. The identification of specific huPrP regions that are crucial for the interaction with Aβ may also contribute to the development of therapeutic strategies that target this interaction.

## Author Contributions

YuZ conceived and designed the manuscript. YuZ, YaZ, and LZ analyzed and collected the literature. WY, YW, and WC collected the literature. YuZ wrote the review. All authors revised the manuscript.

## Conflict of Interest Statement

The authors declare that the research was conducted in the absence of any commercial or financial relationships that could be construed as a potential conflict of interest.
